# Automated identification of the origin of energy loss in nonoriented electrical steel by feature extended Ginzburg–Landau free energy framework

**DOI:** 10.1038/s41598-025-00357-z

**Published:** 2025-07-15

**Authors:** Michiki Taniwaki, Ryunosuke Nagaoka, Ken Masuzawa, Shunsuke Sato, Alexandre Lira Foggiatto, Chiharu Mitsumata, Takahiro Yamazaki, Ippei Obayashi, Yasuaki Hiraoka, Yasuhiko Igarashi, Yuta Mizutori, Sepehri Amin Hossein, Tadakatsu Ohkubo, Hisashi Mogi, Masato Kotsugi

**Affiliations:** 1 Tokyo University of Science, Tokyo, Japan; 2 University of Tsukuba, Tsukuba, Japan; 3 Okayama University, Okayama, Japan; 4 Kyoto University, Kyoto, Japan; 5 Inamori Research Institute for Science, Kyoto, Japan; 6 RIKEN Center for Advanced Intelligence Project, Tokyo, Japan; 7 NIMS, Tsukuba, Japan; 8 Nippon Steel, Chiba, Japan

**Keywords:** Ferromagnetism, Magnetic properties and materials, Theory and computation, Ferromagnetism, Magnetic properties and materials, Characterization and analytical techniques, Imaging techniques, Microscopy

## Abstract

This study presents the automated identification of the complex magnetization reversal process in nonoriented electrical steel (NOES) using the feature extended Ginzburg–Landau (eX-GL) free energy framework. eX-GL provides a robust connection between microscopic magnetic domains and macroscopic magnetic hysteresis using a data science perspective. This method employs physically meaningful features to analyze the energy landscape, providing insights into the mechanisms behind function. We obtained features representing both the microstructure and energy of the domain wall. The causes of iron loss were traced to the original domain structure, through which we could successfully distinguish and visualize the role of pinning as a promoting and resisting factor. We found that the reversal process was governed not only by general grain boundary pinning but also by segmented magnetic domains within the grain. This method revealed the complex interplay between magnetism and metallography and introduced a new means for transformative material design, bridging structures and functions.

## Introduction

Energy loss (iron loss) is a critical magnetic property determining the driving efficiency of motors and is critical for next-generation electric vehicles^1–5^. In soft magnetic materials, energy loss accounts for ∼30% of the total loss observed in motors. In the United States, energy loss in electrical steel represents 4.5% of the total energy loss, contributing to the emission of 40 million tons of carbon dioxide^6,7^. The significant global concern regarding this material is demonstrated by initiatives such as Google’s Little Box Challenge and the reported scientific review article by Science^8^. Nonoriented electrical steel (NOES) is a prominent soft magnetic material used in the stator iron core of various motors. NOES-based motors have a substantial market share (97%), and the global research and development of soft magnets is rapidly accelerating. Improving the overall energy loss of motors is a crucial challenge in the field of magnetics^7,9^.

Regardless, the origin of energy loss incurred during the magnetization reversal process remains unclear. Macroscopic energy loss is defined as the area within a magnetic hysteresis loop. The energy expended during the macroscopic magnetization reversal process depends highly on the complex changes in the microscopic magnetic domain structures. The complex metallographic structures in NOES undergo complicated changes in their magnetic domains^7,10^. The microscopic magnetic domain walls are pinned at the grain boundaries of the metallographic structures, leading to increased coercivity and resulting in macroscopic energy loss. However, these complex magnetic domain structures have been primarily interpreted visually, whereas the underlying mechanism has been only discussed qualitatively and subjectively.

Fundamentally, the correlation between the macroscopic energy loss and microstructure of magnetic domains is promising for further investigation^11–15^. Particularly for inhomogeneous practical materials, the relationship between the function and structure is not fully understood. Most extant physical models are designed for homogeneous systems and have not adequately addressed the complexities of inhomogeneous systems thus far^16,17^. The conventional Steinmetz formula was proven to be inadequate for accurately representing the microstructure of domain walls. Therefore, the origin of energy loss has been an important issue in magnetics for over half a century.

The Ginzburg–Landau (GL) free energy was a useful concept for analyzing the magnetization reversal phenomena in a homogeneous system^18^. Recent progress in data science has led to analyzing inhomogeneous systems using a feature extended GL (eX-GL) model^19–23^. The modern concept of topology, persistent homology (PH), enables the analysis of spatial inhomogeneity in microstructures^20–25^. A principal component analysis (PCA) extracts the essential features hidden in complex data. Using these interpretable analytical methods, the free energy landscape can be drawn in the data space. The microscopic structure and macroscopic function are robustly connected by physically meaningful features, enabling visualization of the cause of energy loss back to the magnetic domain structure. Groundwork regarding simulated magnetic domain data and experimental data on near-ideal thin films has been conducted^19–22^.

In this study, we developed the eX-GL framework for the automated analysis of the complex magnetization reversal process in NOES, which is the actual material including various grain boundaries and defects. Here, “automated” analysis refers to the ability to pinpoint specific factors contributing to iron loss simply by applying interpretable machine learning to magnetic domain structure images and corresponding energy data. Moreover, expert knowledge can deepen the interpretation of the physical phenomena and contextualize the findings. Our research addresses the gap in the existing literature by linking the microscopic structure to the macroscopic function. We employed physically meaningful features to analyze the energy landscape to understand the underlying mechanisms. The study aims to trace the causes of iron loss to the original domain structure, elucidate the role of the domain wall, and uncover the complex interplay between magnetism and metallography. In this context, the term iron loss specifically refers to static hysteresis loss, while dynamic magnetization reversal processes are beyond the scope of this study.

## Results

**Fig. 1:**
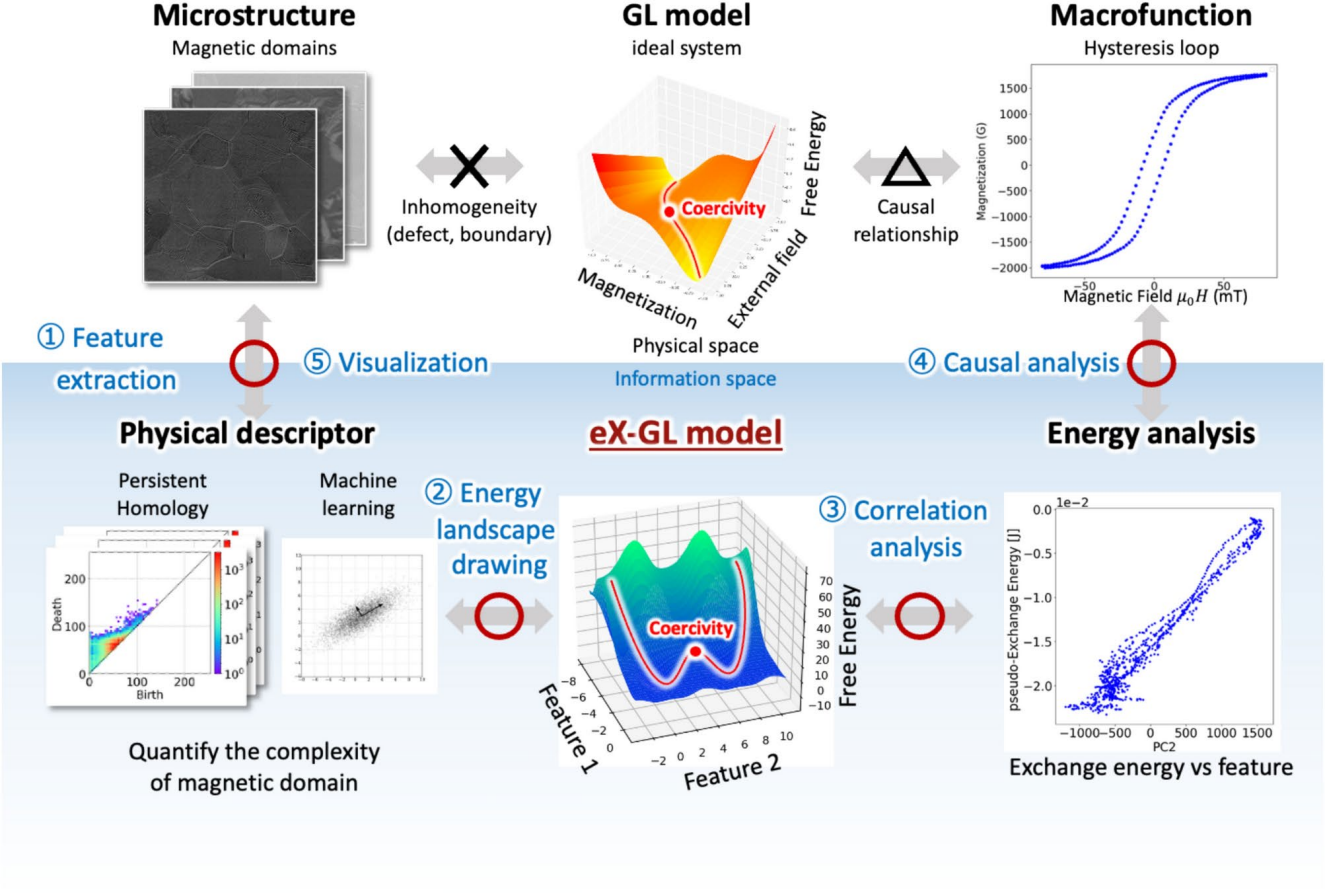
Workflow of the magnetization reversal process analysis using the eX-GL model. The eX-GL model can address the complex inhomogeneity of the structure and establish a connection to the functions from a data science perspective. PH was employed to extract the features of the experimental magnetic domain structures, and dimensionality reduction was performed using highly interpretable machine learning. The energy landscape was drawn in the data space using these physically meaningful features. An energy landscape analysis was performed based on the relationship between the energy and features, which was used to visualize the origin of the macroscopic functions in the original microscopic structure.

Figure 1 presents the workflow of the causal and transformative analyses using the eX-GL model. PH was applied to extract the topological features of the magnetic domain structure as big data, which was obtained using a Kerr microscope; PCA was conducted to reduce the dimensionality. We constructed an eX-GL energy landscape using the obtained features and energy and established the relationship between the microscopic magnetic domain structure and macroscopic hysteresis loop^19–22^. The landscape was analyzed based on the relationship between the features and energy. A comprehensive correlation analysis was performed between the features and physical parameters to design meaningful physical features that contribute to the function. These features can be traced to the original magnetic domain structure across the super-hierarchy. This enables identifying the role of magnetic domain walls and visualizing their behavior in complex magnetization reversal; details are described in the Methods section.

**Fig. 2:**
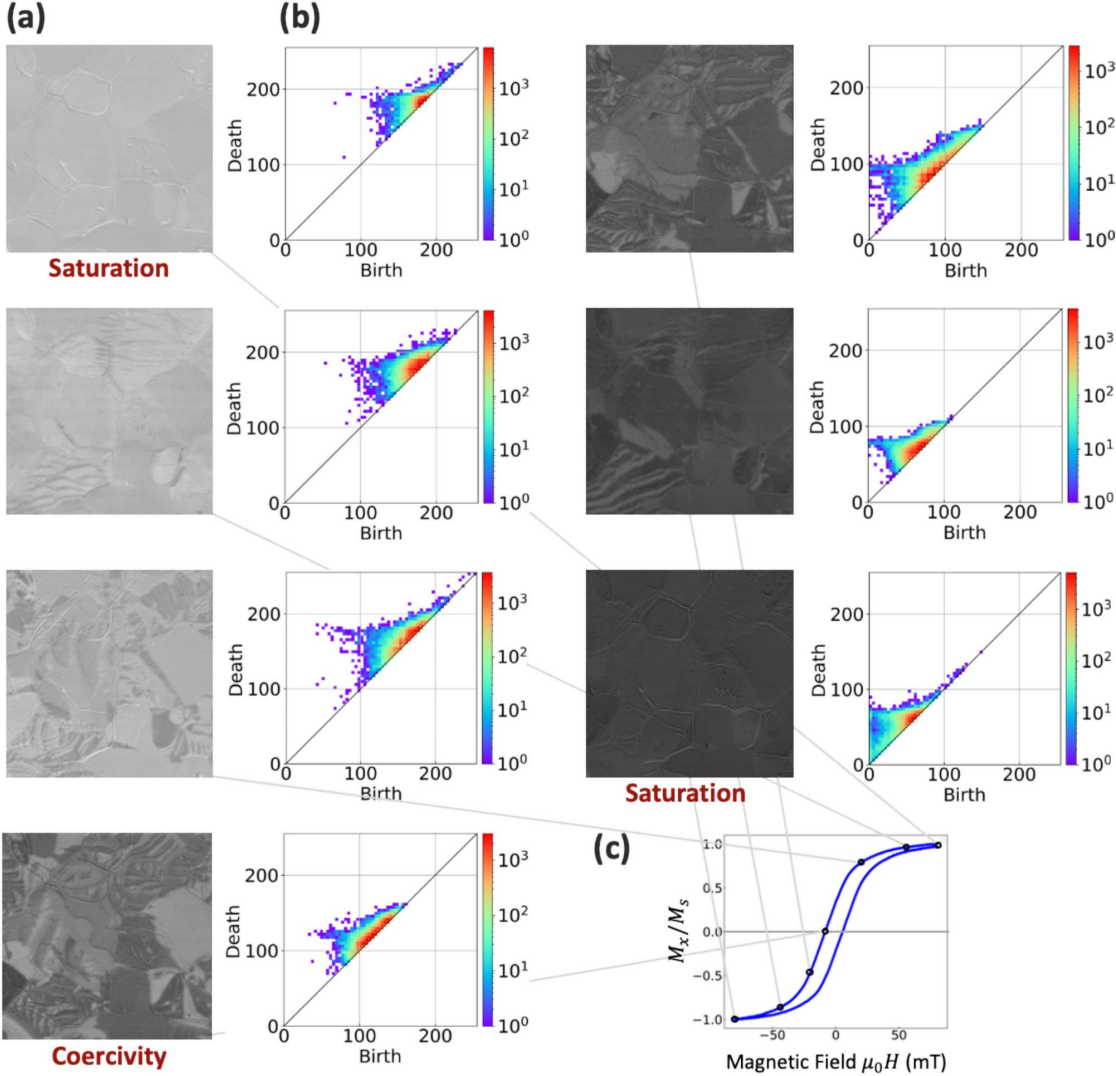
Magnetic domain structures (**a**), the corresponding persistence diagrams (**b**), and magnetic hysteresis loop (**c**). Magnetic domains of NOES were obtained by Kerr microscope. The brightness of each pixel represents the x component of magnetic moment. The magnetic domain structure evolves in a complex manner depending on the applied magnetic field. The persistence diagram, constructed via persistent homology, captures the complex behavior of magnetic domain structures. The Birth and Death axes represent the specific time points in the filtration process at which a topological feature appears and disappears, respectively. The maximum value on these axes is 255, as the input data for the Persistent Homology analysis consists of 8-bit images. The color map indicates the multiplicity of topological features. In the magnetic hysteresis loop, the magnetization on the vertical axis is derived from the average brightness of the magnetic domain structure, and the horizontal axis represents the applied magnetic field. The representative points on the hysteresis curve correspond to each magnetic domain structure and PD. The observed low coercivity shows typical behavior for soft magnetic materials.

The magnetic domain was obtained on commercially available NOES (50H800) using a Kerr microscope (Fig. 2a). The magnetic hysteresis loops showed a continuous change in magnetization depending on the external field (Fig. 2c). Magnetization was sufficiently saturated at ± 80 (*μ*_*0*_*H*) mT, and coercivity was − 11 (*μ*_*0*_*H*) mT; μ_0_ indicates vacuum magnetic permeability.

The corresponding magnetic domain is illustrated in Fig. 2a. The brightness of each pixel represents the magnetic moment projected in the x direction. Continuous, complex changes in the magnetic domain structure were confirmed. Initially, a reversed magnetic domain was generated in the white-saturated state, followed by the growth of variously shaped domains, eventually reaching a black-saturated state.

We employed PH to obtain the structural features of the complex magnetic domain structures (see Methods). A persistence diagram (PD) quantifies the geometric characteristics of structures, including their connections and void shapes. The generator in a PD quantifies microstructural shape information (Fig. 2b). Here, each “generator” (or “PD generator”) corresponds to a point on the Persistence Diagram and is defined by its birth and death values, which are directly linked to brightness levels within the images.

The continuous change in the PD generator distribution depended on changes in the magnetic domain structure. Generators were concentrated at the PD center for coercivity, and the edges for saturation. PDs are useful for characterizing the inhomogeneity of a magnetic domain structure.

Figures 2b show how the distribution of points on the Persistence Diagram is closely tied to the image brightness distribution observed in the magnetic domain images over the magnetization reversal process. Each magnetic domain image thus corresponds to its own Persistence Diagram, which we use as an order parameter that accounts for the system’s microscopic inhomogeneity. The overall width of the distribution on the single Persistence Diagram reflects the range of the distribution of the brightness in a given image, since birth and death values depend on the pixel brightness. Furthermore, images near the coercive region often have a wider brightness distribution, producing more intricate domain structures. The Persistence Diagram effectively captures this added complexity through a broader, more varied spread of points, revealing the diversity of brightness (and thus topological features) that arise in those regions.

### Dimensionality reduction by PCA

We applied the PCA to the PDs to reduce complex high-dimensional datasets to lower dimensions. PCA has the advantage of extracting the intrinsic features of data as eigenvectors, while visualizing trends of data change in a low-dimensional space. It enabled quantifying the complexity and fine structure of the magnetic domain structures and visualizing the complex magnetic reversal process (see methods section). In contrast, conventional methods such as direct image analysis or Fourier transforms can struggle to simultaneously capture features across long-, medium-, and short-range length scales, as described in^21^. By integrating persistent homology (which encodes structural features) and PCA (which highlights dominant modes of variation in the structural feature of the magnetization reversal process), we gain a more comprehensive understanding of how the system’s energy landscape evolves during the magnetization reversal process. This combined approach manages spatial inhomogeneity more effectively than standard techniques.

### Estimation of pseudo-exchange energy

The pseudo-exchange energy was estimated from the magnetic domain structure using the XY model, which analyzes the exchange energy based on the in-plane magnetization (see methods section).

### Feature extended energy landscape

**Fig. 3:**
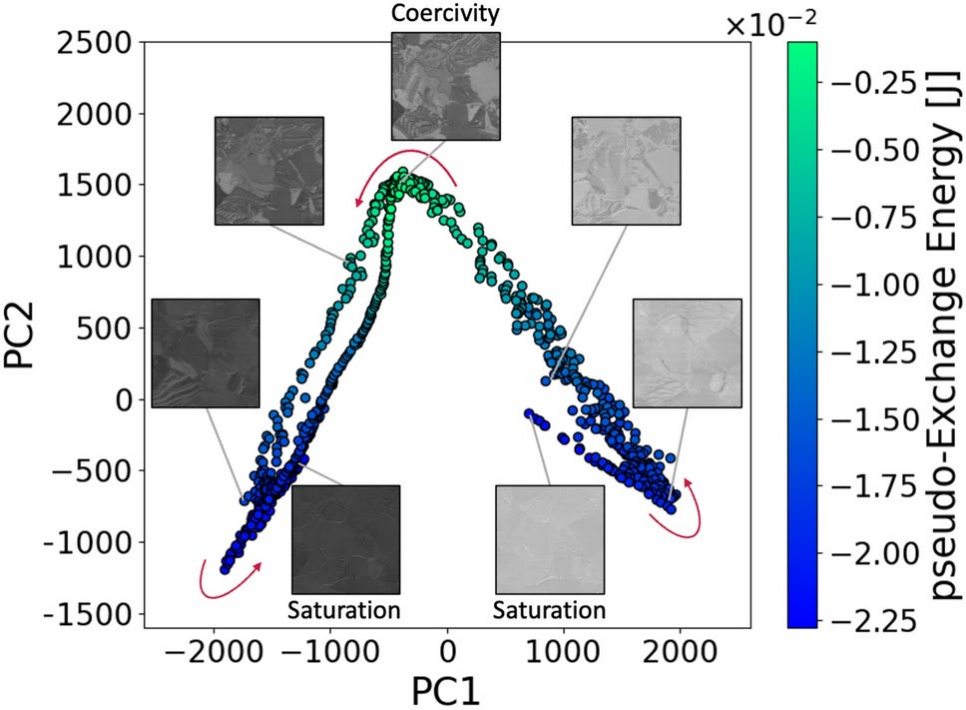
eX-GL energy landscape illustrating the magnetization reversal process in the data space. The distance between the data points corresponds to the change in the magnetic domain structure, and the colors represent the pseudo-exchange energy. The relationship between the structural changes and energy costs is constructed in the data space.

We present the extended energy landscape by visualizing the changes in the magnetic domain structure and energy in the data space (Fig. 3). The horizontal- and vertical-axes represent PC1 and PC2, respectively, as determined by the PCA. The pseudo-exchange energy was color-mapped to each point on the PCA scatter plot. Each data point corresponds to a magnetic domain image. The distance between the data points corresponds to the change in the PDs, which indicates the change in the magnetic domain structures^21^. Thus, Fig. 3 demonstrates the relationship between the magnetic domains and pseudo-exchange energy in the low-dimensional space. The data points and energies exhibit continuous and symmetrical behavior. The central bending corresponds to the coercivity region. PC1 was found to be almost zero, PC2 was the maximum, and the pseudo-exchange energy was the maximum. The pseudo-exchange energy was related to the existence of the magnetic domain wall. The number of magnetic domain walls was the highest at the coercivity as the feature value increased. These results are consistent with those of a previous study^20,21^. Therefore, this study focused on exchange energy to advance the analysis. Our findings confirm the close relationship among the structure, energy, and functions.

## Discussion

**Fig. 4:**
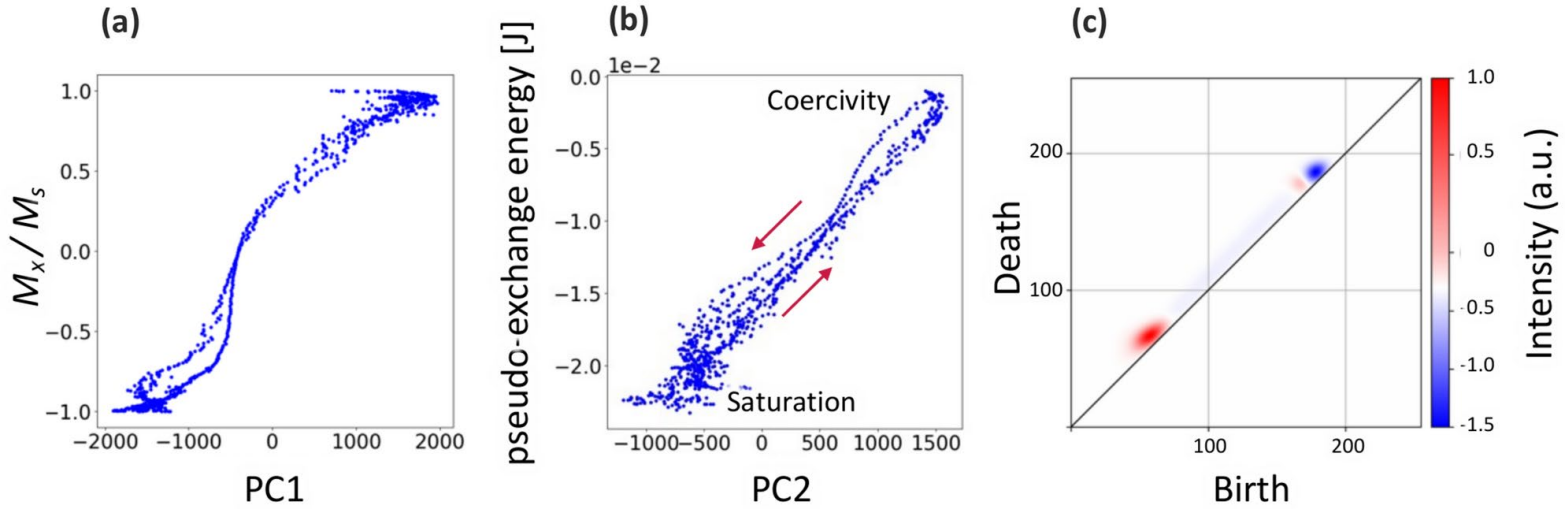
Correlation analysis between the features and physical properties. (**a**) PC1 score versus magnetization demonstrates a monotonous increase. (**b**) PC2 score shows a proportional relationship with the pseudo-exchange energy. (**c**) Element-wise product of the PD vector and PC2 eigenvector. The elements-wise product is the product of each element of the PCA eigenvectors and the PD vector. The obtained vector describes both the domain wall energy and the microstructure of the magnetic domain. In the Persistence Diagram, the Birth and Death axes represent the specific time points in the filtration process when a topological feature appears and disappears, respectively. The maximum value on these axes is 255, determined by the filtration process based on the 8-bit grayscale intensity of the input images. The color map in the Persistence Diagram indicates the intensity of the element-wise product.

We analyzed the correlation between the obtained features and magnetic properties to discuss the mechanism of the magnetization reversal process. The PC1 score demonstrated a monotonous increase in magnetization (Fig. 4a). The PC1 score is a useful explanatory variable for the magnetization. Moreover, PC2 demonstrated a clear proportional relationship with the pseudo-exchange energy, as illustrated in Fig. 4b. Nearly no outliers can be observed in the correlation between PC2 and pseudo-exchange energy. The exchange energy is directly related to the existence of magnetic domain walls. Therefore, PC2 is a robust explanatory variable for the pseudo-exchange energy and the features of the microscopic magnetic domain wall. The obtained PC1 and PC2 are useful features that not only explain the magnetic domain structure but also the magnetic property.

The element-wise product was used to reconstruct the scalar value into vectors. The product of each element of the eigenvector and PD vector was obtained, and a new vector was constructed. The magnetization reversal contribution vector maintained the same physical dimensions as that of the PD vector, and the sum of each component corresponded to a single score of PCA. As indicated above, PC1 and PC2 describe the magnetization and domain walls, respectively (Fig. 4a,b). Thus, the element-wise product enables constructing a feature describing both the physical properties and the microstructure. The detailed procedure is described in the Methods section and can be found in a previous study^22^.

The elements-wise product obtained from the PC2 eigenvector and PD vector is shown in Fig. 4c. The obtained vector describes both the domain wall’s energy and the magnetic domain’s microstructure (Fig. 4c). The value of each element of the vector is either positive, negative, or zero, which affects the value of a single score of PC2. Each element can be seen as a factor that increases or decreases the energy due to the proportional relationship between the PC2 score and pseudo-exchange energy. Using this property enables distinguishing between the increases and decreases in the exchange of energy and visualizing their role in the reversal process.

We visualized the promoting and resisting factors during the magnetization reversal process. The negative components of the product (blue generators) decreased the PC2 score and contributed to the decreased pseudo-exchange energy, as discussed in Fig. 4b. The negative components can be interpreted as the promoting factors for magnetization reversal. Conversely, the positive components (red generators) increase the PC2 score and pseudo-exchange energy, serving as resisting factors for magnetization reversal. Both factors indicate how domain walls behave in terms of the exchange energy. Here, the “promoting” factor indicates higher susceptibility to domain wall motion, whereas the “resisting” factor corresponds to a higher penalty or resistance, as viewed through the exchange energy framework. However, easily moving domain walls do not always coincide with the promoting factor in some cases of this work. That is because that magnetization reversal should ideally be analyzed in terms of all relevant magnetic energies, but our present approach focuses on the exchange energy alone. This focus explains why easily moving domain walls do not always coincide with the promoting factor; however, certain domains, such as the circular ones at 36 mT discussed in Fig. 6f, do align with our analysis. It is also important to carefully note the presence and absence of both factors. There are four possible scenarios derived from whether promoting and resisting factors are present or absent, which Fig. 6a–d illustrates with particular attention to the areas marked by yellow arrows and circles. Notably, the circular magnetic domains described earlier exhibit only the promoting factor. This perspective hopefully provides a useful foundation for understanding domain wall propagation from the standpoint of exchange energy.

This work focuses on the role of magnetic domain walls in the magnetization reversal process, specifically through the lens of the exchange energy. While it is true that all energy contributions should be included for a complete picture, accurately computing each one remains difficult—particularly given the limited resolution of experimental images and the fact that we have only measured the x component of magnetization. Despite these constraints, examining the exchange energy alone offers valuable insight into how domain walls influence magnetization reversal. This is because our study uniquely analyzes inhomogeneous magnetic systems in terms of the exchange energy penalty, providing a foundational step toward a more comprehensive, energy-based understanding.

**Fig. 5:**
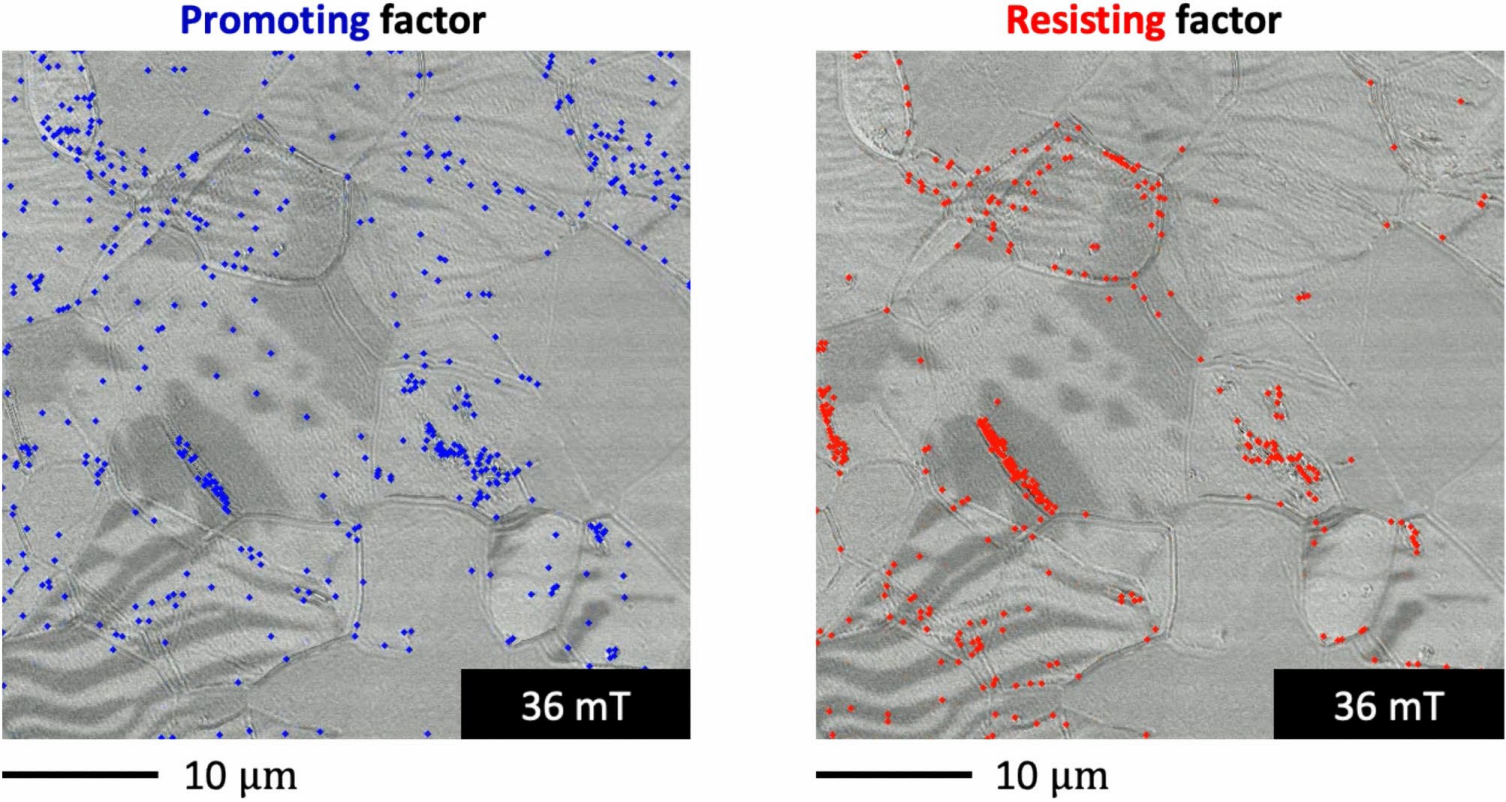
Promoting and resisting factors for the magnetization reversal process visualized using an element-wise product; blue plots indicate the promoting factor in the magnetic domain wall movement, whereas red plots indicate the resisting factor (see supplemental movie).

**Fig. 6:**
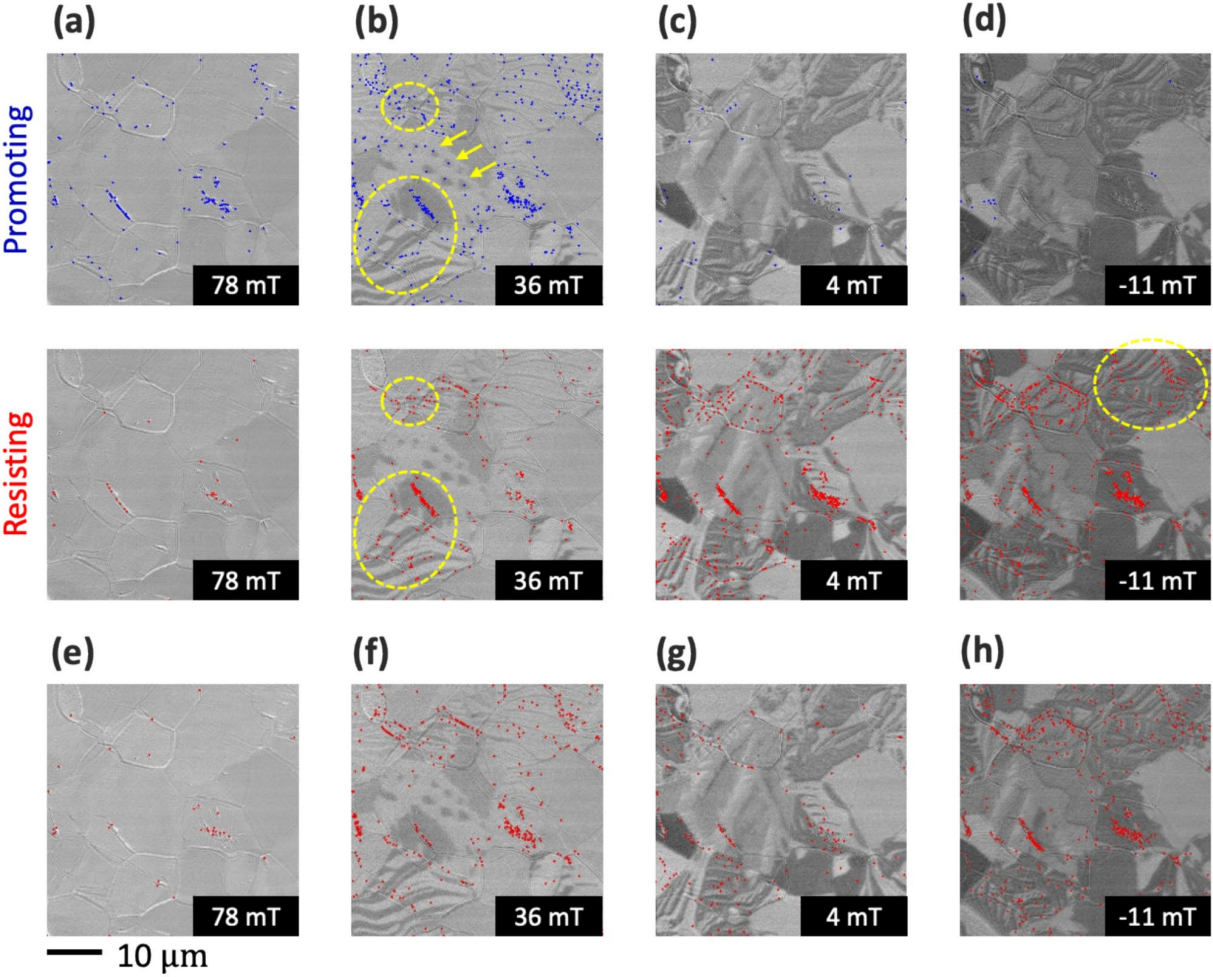
Visualization results of the promoting and resisting factors in typical magnetic fields. (**a**) Initial stage of magnetization reversal at 78 mT. The promoting factors appeared at various locations, providing consistency to the initial stage of the reversal process. (**b**) Subsequent stage of magnetization reversal at 36 mT. The promoting factor was mapped to the circular magnetic domains (arrow), which captured a smooth magnetization reversal. Both the promoting and resisting factors were plotted at the same location (circles), indicating the competition between the increase and decrease of the pseudo-exchange energy. It sufficiently agrees with the general pinning phenomena at the grain boundary. (**c**) Progressive stage of magnetization reversal up to 4 mT. The domain walls with and without generators enable the identification of their contribution and physical role in the magnetization reversal phenomena. (**d**) Coercivity region at − 11 mT. Resistance factors were plotted in the elongated magnetic domains within the grain (circle). These magnetic domain walls hardly move and inhibit magnetization reversal, which sufficiently explains the coercivity phenomenon. (**e**–**h**) Visualization results for PC1. The change in magnetization shows a similar spatial distribution of the resistance factor.

The promoting and resisting factors in the magnetic domain were illustrated using element-wise products and HomCloud, as illustrated in Fig. 5 (see supplemental movie). The blue and red plots indicate the promoting and resisting factors in the magnetic domain wall propagation, respectively. We evaluated the quantity and spatial distribution of the plots to understand the complex pinning mechanism. Pinning is widely known to occur near the grain boundary, resulting in complex changes in the magnetic domain structure. The elements-wise product, obtained from the PC2 eigenvector and PD vector, is not directly influenced by differences between adjacent frames. That is because the images, such as Fig. 6a–d are generated from the element-wise product of each corresponding Persistence Diagram shown in Fig. 4c. Although the PC2 scores do incorporate information about overall image features and background contrast, we have not observed the kind of disruptive noise in Figs. 3 or 4b that would manifest as obvious outliers if background contrast were significantly affecting the analysis.

Furthermore, even when the global brightness changes from one frame to the next, the relative range of pixel intensities within each individual image could remain stable. This consistency also likely explains why variations in background brightness do not substantially alter the visualizations in Figs. 5 and 6. To counter potential noise in individual images, such as scratches or other artifacts, we fine-tuned the weighting function used to transform the Persistence Diagram into its vector form. By doing so, we suppress noise-related generators near the diagonal line, thus reducing their overall influence.

While our approach does not artificially identify points, it can struggle to distinguish magnetic structures like domain walls from non-magnetic grain boundaries if only brightness-based data are available as input data. We can readily address these identified points because we understand why they appear. By explicitly recognizing and excluding them in a reasonable manner, we keep the focus on meaningful magnetic phenomena.

The promoting factors were plotted at the beginning of magnetization reversal at 79 mT. The number of promoting factors ranged from 79 to 4 mT, and decreased from 4 mT to − 30 mT. The promoting factors were distributed on the magnetic domain wall and everywhere in the viewing field. The number of resisting factors was small at external fields from 79 to 48 mT, and increased from 48 mT to − 25 mT. The resisting factors were selectively plotted on the magnetic domain walls. The specific magnetic domain walls contributing to the magnetization reversal process were visualized.

The cause of magnetization reversal is discussed based on the snap shots of the visualization result (Fig. 6). Numerous promoting factors were present at 78 mT, whereas the number of resistive factors was relatively limited, suggesting that the magnetization reversal process was in the initial stages (Fig. 6a). Therefore, the distribution of both the promoting and resisting factors can sufficiently explain the initial stage of magnetization reversal.

As the magnetization reversal progressed to 36 mT, distinctive circular magnetic domains appeared, where only the promoting factor was plotted (indicated by the arrows in Fig. 6b). These circular magnetic domains gradually expanded and connected with other magnetic domains as magnetization reversal progressed, confirming the role of the promoting factor. Furthermore, plotting only the promoting factors with these domains indicated that these domains smoothly expanded without obstacles owing to the pinning effect. Promoting factors appeared at various locations both at the boundary and within the grains. The occurrence of reverse magnetic domains in soft magnetic materials is generally regarded as a stochastic event, which is consistent with conventional explanations.

Both the promoting and resisting factors were plotted at the grain boundaries (indicated by circles). As illustrated in Fig. 4, the promoting and resisting factors respond to increases or decreases in the pseudo-exchange energy. The concurrent plotting of both factors at the same location indicates that the promoting and resisting magnetic walls compete with one another. This result sufficiently agrees with the general pinning phenomenon, leading to an automatic analysis of the pinning event. The pseudo-exchange energy, which exhibits spatial fluctuations at the grain boundary, contributes to the pinning phenomenon. Note, the incorporation of automated pinning visualization provides substantial advantages in the functional analysis.

The magnetization curve in the hysteresis loop exhibited a pronounced slope within the 48–4 mT range. The magnetic domain wall is believed to contentiously propagate. The analysis shows plotting of both magnetic domain walls with and without generators (Fig. 6c). Although the magnetic domain walls appeared to be visually similar, their roles in magnetization reversal varied. Our method can distinguish and visualize the types of magnetic domain walls, which have been difficult to manually identify.

The coercivity region is at − 11 mT, where the resisting factor dominates, whereas the promoting factor is rarely plotted (Fig. 6d). As previously indicated, the resisting factor correlates with the pseudo-exchange energy and inhibits magnetic wall propagation. The resisting factors were plotted in the elongated magnetic domains within the grain (indicated by a circle), and the magnetic domain wall propagation associated with the external magnetic field was apparently not a smooth process. These magnetic walls inhibit magnetization reversal, which explains the coercivity phenomenon. We successfully extracted information that was previously undetectable by the naked eye.

In summary, the eX-GL model can distinguish the physical role of the domain walls and visualize their distribution onto an image. The location of the promoting factor alone indicated a smooth domain-wall propagation without pinning. The coexistence of both factors indicates that the promoting and resisting factors based on the exchange of energy compete, thereby identifying the location of the pinning phenomenon. In locations with only the resisting factor, the segmented magnetic domain structure inhibited the propagation of the domain wall and contributed to the coercivity. Magnetic domain walls without both factors only have a trivial contribution to magnetization reversal. This result indicates that the grain boundary and intragrains exhibit disparate mechanisms and contributions to coercivity, enabling us to illustrate the cause and location of magnetization reversal using a physical interpretation.

To confirm the PC2 result, we constructed the element-wise product vector from the PC1 score and performed visualization (Fig. 6e–h). The PC1 score correlates with the magnetization, representing both the magnetization and microstructure. The effective field was obtained by summing each vector using the differential vector of PC1^22^. As the local region of the image changes even when the PC1 score monotonically changes, constructing the element-wise product from the differential vector enables a detailed analysis of the changes in the PC1 score. The PC1 score monotonically increases with magnetization, which is the order parameter in the conventional Landau model; thus it can be considered as a natural extension of the Landau theory (see Methods section).

At 78 mT, a few red generators were observed at the grain boundaries (Fig. 6e), suggesting a high energy cost for magnetization reversal at the grain boundaries, which aligns with the previous discussion. At 36 mT (Fig. 6f), red generators were scarce around the circular magnetic domains, indicating limited exchange energy costs, whereas they were more prevalent at the grain boundaries and around the magnetic domain walls, reinforcing the previous discussions regarding pinning. At 4 mT (Fig. 6g), red generators were mostly at the grain boundaries, indicating a higher energy cost and confirming pinning as the primary contributor to energy loss. At − 11 mT (Fig. 6h), red generators were plotted across various locations, especially at grain boundaries, indicating that additional energy is required to propagate the domain wall across the coercive region.

The PC1 visualization results (Fig. 6e–h) present a similar distribution as the resisting factor shown in (Fig. 6a–d). The magnetization change location corresponded to the domain-wall energy barrier, reinforcing each other. These results multidirectionally identified the origin and location of the pinning phenomenon based on the physically meaningful features.

We successfully identified the origin of energy loss during a complex magnetization reversal process in NOES. This method may offer a distinctive framework for examining the spatial inhomogeneity in a range of materials. Compared to the Landau theory, which employs magnetization as an order parameter, the eX-GL model employs shape features as order parameters. As the features are positively correlated with the physical quantities, eX-GL is mathematically consistent with the conventional Landau theory. This study addressed static changes in the magnetic domain structure in inhomogeneous systems. However, there are numerous topics for future investigations, including dynamic processes, thermal effects, and multiphysics, within the context of thermodynamic concepts. In the future, we anticipate that automated analysis of the microstructures of various materials will become standard practice, providing invaluable support to material scientists and enabling the extraction of previously inaccessible information. The integration of data science with advanced microscopic analysis is poised to usher in a paradigm shift, moving beyond mere observations for the formulation of the material design. Additionally, rational functional material designs and the detection of unique phenomena are expected to emerge, which can significantly affect the significance of the measurement.

## Conclusion

We automatically analyzed the complex magnetic domain structures that interacted with the actual metallographic structure by employing the eX-GL model. Visualization of the competitive magnetic wall propagation in the pinning phenomena was achieved using physically meaningful features. The competitive region was found to be mainly distributed at the grain boundary, and the pseudo-exchange energy plays a role in the pinning mechanism. This approach enabled the extraction of information that would otherwise have been difficult to discern via visual inspections and enables the automatic identification of the origin of energy loss in terms of the mechanism and location. Furthermore, it provides guidelines for the material design and future developments.

## Methods

### Sample

The sample was a nonoriented electromagnetic steel sheet containing several grain boundaries and defects, was composed of Fe-Si, and had a typical grain size of approximately 15 $\mu \text{m}$. Sample grade is 50H800. Typical iron loss value is W15/50 = 6.30 W/kg (W15/50: Iron loss at 1.5 T, 50 Hz). Original thickness is 0.5 mm. For Kerr measurement, sample size was modified as 10 mm × 10 mm × 0.204 mm. The sample was initially thinned from 0.5 mm to 0.22 mm using polishing papers with grit sizes No. 400, 1000, and 1500. Subsequently, it was further polished using a polishing machine with fine diamond abrasive particles (6 µm and 1 µm in diameter; DR-Spray, Struers) to achieve the final thickness and obtain a mirror-like surface. The sample was then cleaned with alcohol and dried using a blower. Finally, it was annealed at 700 °C to relieve internal stress and further flatten the surface.

### Experiments

Images of the magnetic domain structure were acquired using a Kerr microscope. The longitudinal polar-optic Kerr effect (in-plane) was used as the optical configuration. The field of view was 44.1 × 44.1 $\mu \text{m}$. The external magnetic field was quasistatically scanned from − 80 mT to + 80 mT with a step size of 0.04 mT for the in-plane direction. A total of 800 images of the magnetic domain structures were sequentially recorded for each hysteresis loop. The magnetic domain contrast was recorded in a 16-bit grayscale. The exposure time was set to 0.1 s to sufficiently exploit the dynamic range of the CCD. The recorded image was 1344 × 1066 pixels in size, with 672 × 672 pixels at the center of the image used for the analysis. The brightness of each pixel represents the magnetic moment projected in the x direction. White pixels correspond to the rightward magnetic moments on the image, and black pixels correspond to those on the left.

### Data preprocessing

To mitigate the noise caused by the microscopic scratches on the surface, singular value decomposition (SVD) was applied. SVD decomposes matrix X, representing the image data, into singular values and their corresponding vectors. The dominant singular values (e.g., first, second, etc.) and their corresponding vectors represent the common components across frames, whereas the less significant singular values represent frame-specific variations.

The dataset was separated into components attributed to surface-polishing scratches (the common components) and those corresponding to magnetic domain structures (the structural changes). The first two components were excluded as noise, and the remaining components were used in the data analysis. The optimal number of components was determined by testing different values and visually evaluating the magnetic domain structure. The image brightness was then accurately corrected using the magnetization obtained with a vibrating sample magnetometer.

### PH analysis

**Fig. 7:**
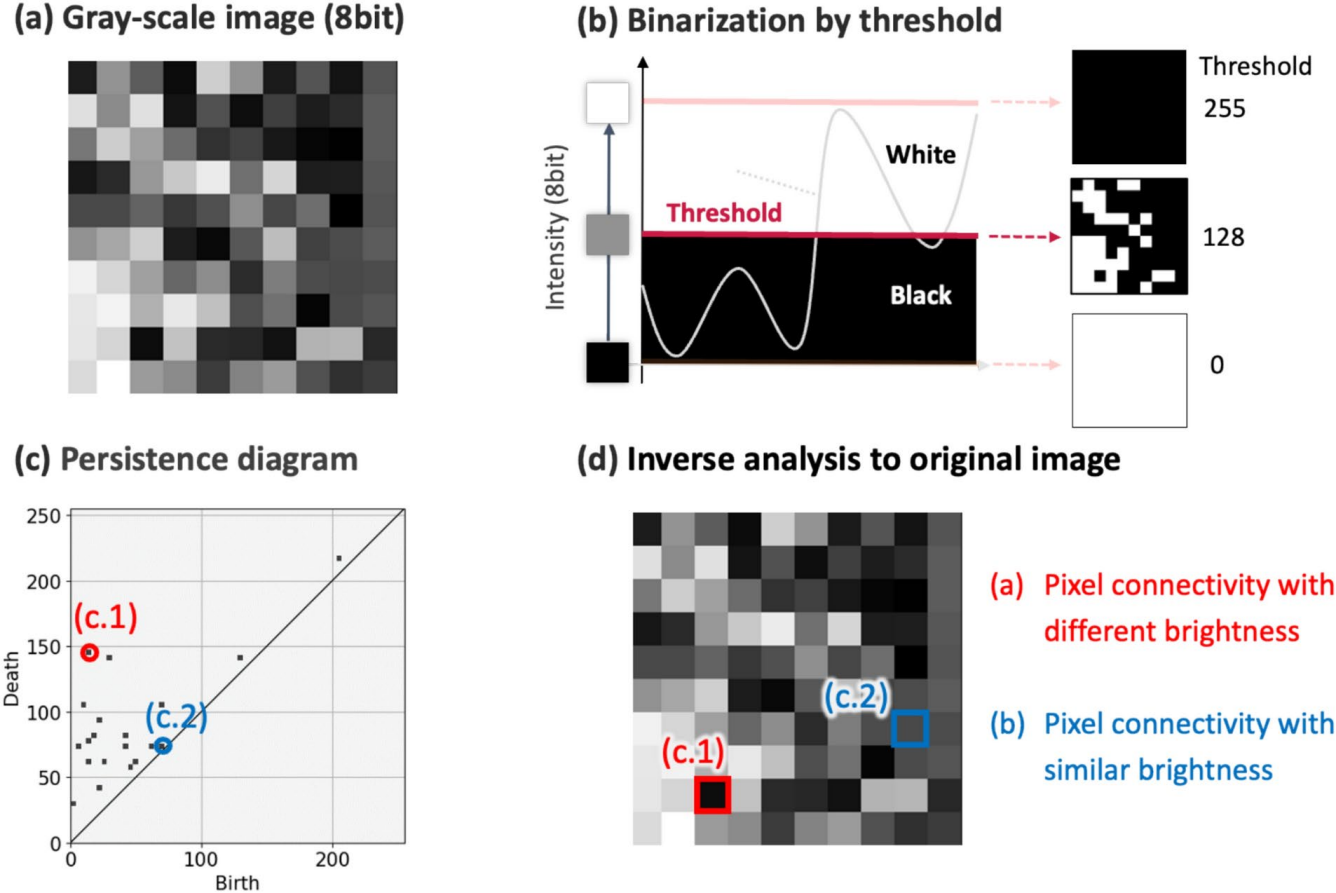
Schematic sequence of the feature extraction from a fine structure using a zero-dimensional persistence diagram (PD0) based on the zero-dimensional persistent homology (PH0). (**a**) 8-bit grayscale image as the input data. (**b**) Binarized images at thresholds of 255, 128, and 0. (**c**) PD0 extraction from the grayscale image. While continuously changing the threshold value (Filtration), “birth” is recorded when a new connected component appears, and “death” is recorded when the connected component merges with another component. (d) Relationship between the generators and original image in PH0. (c.1) and (c.2) can be returned to the original image.

PH is a powerful topological concept that quantitatively describes the structural features (size, shape, fluctuation, and connectivity) of holes and islands (Fig. 7). We used PH to identify the features of the magnetic domain structure. PH quantifies the geometric characteristics, including the connection of the structures and shape of the holes^21,22,24,25^. Using grayscale filtration, we examined an image by assessing the brightness and position of each pixel, and obtained a PD, which describes the geometric features of a grayscale image and quantifies the microstructural shape information. In addition, integrating the PD with machine learning enables correlating the shapes with the physical properties.

The fine structure can be quantitatively evaluated. This method enabled quantifying the complexity of the fine structures in grayscale images by evaluating the connectivity between the pixels while varying the threshold.

In this analysis, an 8-bit grayscale image is used as input data, as shown in Fig. 7a. The image is analyzed through a filtration process, where the threshold value, directly related to image brightness, is gradually increased, allowing us to record the emergence and disappearance of topological features at each stage (Fig. 7b).

The extracted Persistence Diagram (Fig. 7c) provides a topological summary of the image, where the Birth and Death axes represent the specific points in the filtration process at which a topological feature first appears and eventually disappears. In addition to these coordinates, the lifetime, defined as the vertical distance from the diagonal line, quantifies the robustness and significance of each topological feature. Features with a long lifetime are considered robust and meaningful, representing essential structural characteristics, while those with a short lifetime may still provide valuable topological insights but are more susceptible to noise, reflecting small variations in brightness between adjacent pixels.

Furthermore, we execute the inverse analysis from specific generators (key topological features) in the PD to the original input data (Fig. 7d). This approach allows us to identify and interpret the physical meaning of topological features extracted from the image. In Fig. 7d, the highlighted generators (c.1) and (c.2) illustrate how persistent homology can be leveraged to correlate microstructural information with physical properties.

The input images were converted to 8-bit grayscale for the PH analysis. In this analysis, the PD was generated using zero-dimensional subfiltration and superfiltration^24,25^. The grid interval was in the range of {(b,d) ∈ [0,0] × [255,255]}, with a weighting function $\text{arctan}\left(\text{c }\bullet {\left(\text{lifetime}\right)}^{\text{p}}\right)$ consisting of the arctangent function and a power exponent (c = 0.01, p = 3). The Gaussian filter σ was set to 5.

Vectors of the subfiltration PD and superfiltration PD were obtained, which were combined to create features (65,536 dimensions) using Persistence Image^25^. This vectorization was performed on 795 images corresponding to a hysteresis loop, and these features were combined to obtain a feature matrix of (65,536 × 795). HomCloud 3.5 was used for the PH analysis and visualization^24,25^.

### PCA

PCA was applied to the high-dimensional data, which were reduced to two dimensions. PCA has the advantage of extracting the intrinsic features of data as eigenvectors, while visualizing trends in low-dimensional space and the distances between intricate datasets. The physical parameters can be analyzed without requiring complicated distance conversions because the eigenvectors are orthogonal to one another and the Euclidean distance between the data is preserved^26^. The combination of PH and PCA enabled quantifying the complexity and fine structure of the magnetic domain structures and visualizing the complex magnetic reversal process. PCA is a method used for reducing the dimensionality of high-dimensional vectors $\left\{{x}_{i}|{x}_{1}^{i}, \dots , {x}_{p}^{i}\right\}$ to low-dimensional vectors $\left\{{y}_{i}|\left({y}_{1}^{i}, \dots , {y}_{m}^{i}\right), m<p\right\}$.

The eigenvector ${\varvec{W}}=\left\{{w}_{1}, \dots , {w}_{i}\right|{w}_{i}=({w}_{1},\dots , {w}_{p})\}$ is generated, and the high-dimensional data are reduced to a lower dimension as follows (Eq. (1)), retaining as much variance as possible:

1 $$\begin{array}{c}y=Wx\end{array}$$

$$\left({w}_{1}^{2}+{w}_{2}^{2}+\dots +{w}_{i}^{2}=1, x=\left\{{x}_{1}, \dots , {x}_{n}\right\}, y=\left\{{y}_{1}, \dots , {y}_{n}\right\}\right).$$

PCA was applied to obtain eigenvectors that were orthogonal to one another; the magnetic domain structural changes can be discussed in terms of the Euclidean distance. The (65,536 × 795) feature matrix was dimensionally reduced (2 × 795). Machine learning was performed using Python 3.7 with the scikit-learn library^26^. PC1 and PC2 were obtained as eigenvectors with contributions of 58.4% and 18.6%, respectively. These values are reasonable relative to those obtained in our previous studies^20,22,27^. PC1 and PC2 are reliable vectors representing the changes in the magnetic domain structure and enabling effective dimensionality reduction.

### Analysis of the pseudo-exchange energy

The pseudo-exchange energy (Eq. (2)) was estimated using the magnetic domain structure image based on the XY model^28^:

2 $$\begin{array}{c}{E}_{exch}= -{\sum }_{i,j}{{\varvec{M}}}_{{\varvec{i}}}\bullet {{\varvec{M}}}_{{\varvec{j}}},\end{array}$$

where ***M*** is the mean magnetic moment per pixel. The exchange interaction was calculated as the inner product of the magnetic moments. The exchange energy decreased in parallel configurations of the neighboring magnetic moments and increased in antiparallel configurations. We used the second-nearest-neighbor pixels based on a previous study. The in-plane pseudo-exchange energy was estimated by multiplying it with the exchange-stiffness coefficient. Assuming that the sample was sufficiently thin and the magnetic domain structure was consistent in the z-direction, the pseudo-exchange energy was estimated by multiplying the thickness^20^, where *L*[μm] is the thickness of the sample film (204[μm]) and *A*[J/m] is the exchange-stiffness coefficient (${10}^{-11}$ [J/m])^29^.

### Analysis of the magnetization reversal phenomena

The correspondence between the features and energy was examined for the causal analysis. The comprehensive correlation between the feature values and physical parameters revealed that PC2 had a good correlation with the pseudo-exchange energy. We then constructed an element-wise product using the eigenvectors of PC2 and the PD vectors^30^. The magnetization reversal contribution vector ${{\varvec{v}}}_{\mathbf{c}\mathbf{o}\mathbf{n}\mathbf{t}\mathbf{r}\mathbf{i}\mathbf{b}\mathbf{u}\mathbf{t}\mathbf{i}\mathbf{o}\mathbf{n}}$, as defined in Eq. (3), was obtained from the ith magnetic domain image.

3 $$\begin{array}{c}{\mathbf{v}}_{\mathbf{c}\mathbf{o}\mathbf{n}\mathbf{t}\mathbf{r}\mathbf{i}\mathbf{b}\mathbf{u}\mathbf{t}\mathbf{i}\mathbf{o}\mathbf{n}}={\mathbf{PD}}_{\mathbf{i}}\circ {\mathbf{W}}_{\mathbf{P}\mathbf{C}2}= {\mathbf{PD}}_{\mathbf{i}}\circ \left({\mathbf{w}}_{1},{\mathbf{w}}_{2},\dots ,{\mathbf{w}}_{\mathbf{p}}\right).\end{array}$$

The positive and negative components of the element-wise product were used to identify the functions of the magnetic domain walls. We performed an inverse analysis using the element-wise product and visualized the magnetic domain walls contributing to the magnetization reversal process.

Furthermore, we conducted an energy analysis based on the effective field, as indicated in Eq. (4).

4 $$\begin{array}{c}{H}_{ef{f}_{exch}}= -\frac{\partial {E}_{exch}\left(M\right)}{\partial M}= -\frac{\partial {E}_{exch}}{\partial \text{PC}1}\bullet \frac{\partial \text{PC}1}{\partial M}.\end{array}$$

A single score of the $PC1$ component can be decomposed, as shown in Eq. (5).

5 $$\begin{array}{c}\text{PC1}= {\mathbf{PD}}_{\mathbf{v}\mathbf{e}\mathbf{c}\mathbf{t}\mathbf{o}\mathbf{r}}\bullet {\mathbf{W}}_{\mathbf{P}\mathbf{C}1}={\sum }_{\text{k}=1}^{\text{n}}\left(\mathbf{P}{\mathbf{D}}_{\text{k}}\circ {{\mathbf{w}}_{\mathbf{P}\mathbf{C}1}}_{\mathbf{k}}\right)= {\sum }_{\text{k}=1}^{\text{n}}\text{PC}{1}_{\text{k}}\end{array}$$

Equation (4) can be converted into Eq. (6) by submitting Eq. (5).

6 $$\begin{array}{c}{H}_{ef{f}_{exch}}= -{\left(\frac{\partial \text{PC}1}{\partial {E}_{exch}}\right)}^{-1}\bullet \frac{\partial \text{PC}1}{\partial M}=-{\left(\frac{\partial \text{PC}{1}_{0}}{\partial {E}_{exch}}+ \frac{\partial \text{PC}{1}_{1}}{\partial {E}_{exch}}+ \frac{\partial \text{PC}{1}_{2}}{\partial {E}_{exch}}+\dots +\frac{\partial \text{PC}{1}_{\text{n}}}{\partial {E}_{exch}}\right)}^{-1}\bullet \left(\frac{\partial \text{PC}1}{\partial M}\right).\end{array}$$

We can trace back each $\text{PC}{1}_{\text{k}}$ component to the magnetic domain image using the Homcloud function. Overall, $\frac{\partial \text{PC}1}{\partial M}$ and the difference of the pseudo-exchange energy are approximately positive from the saturation to the coercive region. The negative value of $\partial \text{PC}{1}_{\text{k}}$ contributes to increasing the effective field of the pseudo-exchange energy. Namely, the negative components correspond to the energy cost of the magnetization reversal. Energy stress and cost can be visualized and analyzed using these energy analyses.

## Supplementary Information

Supplementary Video 1.

## Data Availability

The data supporting the results of this study are available from the corresponding author upon reasonable request.
